# Inverse Correlation of Superoxide Dismutase and Catalase with Type 2 Diabetes among Rural Thais

**DOI:** 10.3390/nu15092071

**Published:** 2023-04-25

**Authors:** Natnicha Promyos, Pornpimol Panprathip Phienluphon, Naruemon Wechjakwen, Jirayu Lainampetch, Pattaneeya Prangthip, Karunee Kwanbunjan

**Affiliations:** 1 Department of Tropical Nutrition and Food Science, Faculty of Tropical Medicine, Mahidol University, Bangkok 10400, Thailand; natnicha.prm@mahidol.ac.th (N.P.); pattaneeya.pra@mahidol.ac.th (P.P.); karunee.kwa@mahidol.ac.th (K.K.); 2 Faculty of Public Health, Nakhon Ratchasima Rajabhat University, Nakhon Ratchasima 30000, Thailand; naruemon_w@hotmail.com; 3 Department of Nutrition, Faculty of Public Health, Mahidol University, Bangkok 10400, Thailand; jirayu.lai@mahidol.ac.th

**Keywords:** antioxidant enzymes, SOD, CAT, vitamin A, vitamin E, T2D

## Abstract

Oxidative stress contributes to defective antioxidant defenses, which may lead to type 2 diabetes (T2D). This study aimed to elucidate the T2D risks and antioxidant defenses by investigating the superoxide dismutase (SOD), catalase (CAT), vitamin A, and vitamin E status. We observed 102 participants aged 35–66 years from Sung Neon, Nakhon Ratchasima, Thailand. The blood samples were collected to measure the SOD, CAT, vitamin A, and vitamin E concentrations. The SOD and CAT activities were inversely associated with T2D risk. When compared with participants in the highest quartile of SOD and CAT, those in the lowest quartile for T2D risk obtained multivariable-adjusted odds ratios of 4.77 (SOD: 95% confident interval CI, 1.01–22.40; *p* = 0.047) and 4.22 (CAT: 95% CI, 1.07–16.60; *p* = 0.039). The possible influencing factors (e.g., physical activity, total cholesterol, and triglyceride) might mediate the association of SOD and CAT with T2D risk. Meanwhile, the relationship between vitamin A and vitamin E concentrations and T2D risk was insignificant. In conclusion, lower concentrations of antioxidant enzyme activity (SOD and CAT) may be an additional risk factor for T2D.

## 1. Introduction

The incidence of diabetes mellitus has been increasing worldwide. Type 2 diabetes (T2D) accounts for 90% of all diabetes cases, which are particularly seen in middle-aged individuals [1]. According to the Bureau of Noncommunicable Diseases, approximately 941,222 per 100,000 population were diagnosed with T2D in Thailand in 2018. Nakhon Ratchasima, located in Northeast Thailand, is one of the five leading cities in the country with the highest number of diabetes cases (41,819 persons per 100,000 population) [2]. Oxidative stress (OS) might be one of the major risk factors of early-onset T2D and the development of diabetic complications [3,4]. OS is associated with increased reactive oxygen species (ROS) production and decreased efficiency in antioxidant systems [5]. T2D is reportedly significantly related to OS, and persistent long-term hyperglycemia can lead to excessive ROS formation in patients with diabetes [6].

The common defense mechanism against ROS consists of enzymatic (superoxide dismutase (SOD) and catalase (CAT)) and nonenzymatic (vitamins A and E) antioxidants [7]. Antioxidant defenses in blood can be evaluated to predict the risk for T2D and diabetic complications [8]. SOD is a first-line antioxidant mechanism against ROS. It scavenges superoxide anion into hydrogen peroxide and oxygen, which are further broken down into water and oxygen by CAT [9]. SOD is reportedly associated with T2D by improving OS induced by hyperglycemia [10]. A decline in SOD concentrations may increase the susceptibility to OS in patients with T2D [4]. Another important antioxidant enzyme is CAT, which degrades hydrogen peroxide into oxygen and water [4]. CAT deficiency is associated with increased T2D risk, contributing to β-cell function failure, given that CAT helps protect β-cells from damage by ROS [4]. The literature reviews indicated that low CAT activities in patients with T2D are consistently linked to T2D development and that hyperglycemia leads to the downregulation of *CAT* expression [4,11]. However, previous studies have shown conflicting results on antioxidant enzyme status. For instance, both SOD and CAT concentrations were reduced, unchanged, or increased in patients with T2D when compared with those in the controls [3,6].

Vitamins play important roles in biological systems. Vitamin A (retinol) is an essential fat-soluble vitamin with radical scavenging properties and is required for β-cell maintenance [12]. Patients with T2D reportedly have lower vitamin A levels than healthy participants [13]. In addition, patients suffering from T2D with malnutrition have vitamin A deficiency, and dietary vitamin A intake might improve β-cells with glucose-stimulated insulin secretion [14,15]. Vitamin E (α-tocopherol) is an important natural lipophilic antioxidant. It helps ameliorate high blood glucose concentrations [16]. Vitamin E levels are also reportedly lower in patients with T2D than in those without T2D; supplementation of this vitamin could delay diabetic complications because it aids in reducing ROS formation [14,16]. However, other studies reported otherwise. Some demonstrated that the levels of vitamins A and E in patients with T2D are reportedly lower, higher, or the same as those of the controls [6].

Although some studies already examined antioxidant profiles, the results on the status of antioxidant defenses in patients with T2D are still ambiguous, and the measurements of circulating antioxidant vitamins remain scarce. Therefore, this study aimed to assess and clarify the association of integrated antioxidant parameters of enzymatic and nonenzymatic processes, including SOD, CAT, vitamin A, and vitamin E, with the risk of T2D, especially in Nakhon Ratchasima, Thailand.

## 2. Materials and Methods

### 2.1. Study Population

This cross-sectional study consisted of 102 individuals aged 35–66 years living in rural areas in the Sung Neon District, Nakhon Ratchasima Province. This study is part of the project “Prospective study of diet, Lifestyle, Insulin Resistance, Inflammatory Markers, and Risk of Developing Type 2 Diabetes Mellitus in Rural Thais”. The demographic characteristics of the participants were reviewed. We included patients diagnosed with T2D according to the following American Diabetes Association guidelines: fasting blood glucose level (FBG) ≥ 126 mg/dL, 2 h blood glucose level (2 h BG) ≥ 200 mg/dL, or glycated hemoglobin (HbA1c) ≥ 6.5%. On the other hand, for the control group subjects that participated in this study the following levels were included: FBG ≤ 125 mg/dL, 2 h BG ≤ 199 mg/dL, or HbA1c ≤ 5.0%. The ethics review committee of the Faculty of Tropical Medicine in Mahidol University approved the study protocol (TMEC19-089). All participants provided written informed consent.

### 2.2. Anthropometric Measurements

We measured participants’ height and weight and calculated their body mass index (BMI). In addition, systolic blood pressure (SBP) and diastolic blood pressure (DBP) were monitored using an electronic blood pressure machine. We also measured their abdominal circumference (AC), waist circumference (WC), and hip circumference (HC) and calculated the waist-to-hip ratio (WHR). A body composition analyzer (HBF-375, Omron Healthcare, Kyoto, Japan) was used for identifying the percentage of body fat, visceral fat, trunk fat, and muscle mass.

### 2.3. Biochemical Analysis

The blood samples were collected after an overnight fast of at least 12 h to measure the FBG, 2 h BG, HbA1c, total cholesterol (TC), high-density lipoprotein cholesterol (HDL-C), and triglyceride (TG) levels. The low-density lipoprotein cholesterol (LDL-C) level was calculated using the Friedewald equation, and insulin resistance and β-cell function were estimated using the Homeostatic Model Assessment for Insulin Resistance (HOMA-IR) and Homeostatic Model Assessment for β-cell function (HOMA-β) [17].

### 2.4. Antioxidant Enzyme Measurements

Serum SOD activities were determined using a commercially available kit (Cat. Ab65354; Abcam, Cambridge, UK) according to the manufacturer’s instructions. The inhibition of SOD activity was colormetrically evaluated by monitoring on a 96-well microplate reader (Biotek Synergy H1; Agilent, Santa Clara, CA, USA) with a 450 nm absorbance. The obtained values of SOD activity are expressed as the percentage of inhibition. In analyzing the serum CAT activity, we used a commercial assay kit (Cat. Ab83464; Abcam, Cambridge, UK). The absorbance was recorded colormetrically at 570 nm with a 96-well microplate reader. The results are expressed as nmol/min/mL.

### 2.5. Vitamin Measurements

Serum vitamins A and E concentrations were measured using reverse phase HPLC (Immundiagnostik AG, Waldkirch, Germany). Using an ultraviolet detector, we detected vitamins A and E at 325 and 292 nm, respectively, using the HPLC Dionex UltiMate 3000 (Thermo Scientific; Waltham, MA, USA). The results are expressed as mg/L.

### 2.6. Dietary Assessment

Dietary information was assessed using a semiquantitative food frequency questionnaire (semi-FFQ). The trained staff interviewed the participants according to the semi-FFQ and asked them how often each of the food types were consumed. The estimated dietary intake was calculated using the NutriSurvey software version 2007 (SEAMEO-TROPMED RCCN-University of Indonesia).

### 2.7. Statistical Analysis

The data are presented as median (min, max) for the continuous variables and as number (percentages) for the categorical variables. Between the T2D and control groups, the continuous and categorical variables were compared using the Mann–Whitney’s *U* test and Pearson’s chi-squared test, respectively. The independent association between the antioxidant defenses and the T2D risk was determined using logistic regression analyses without or with the adjustment of any confounding factors. The odds ratios (ORs) for the T2D incidence and the 95% confidence intervals (95% CIs) were calculated for the quartiles of the levels of the antioxidant defenses (SOD, CAT, vitamin A, and vitamin E) to predict the T2D risk. The highest quartile of the antioxidant defenses was indicated as the reference category.

We estimated the crude models and adjusted the ORs of each of its components. The first model (model 1) was adjusted for any potential confounding factors, including age (years, continuous) and sex (male, female). Model 2 was based on model 1 and was additionally adjusted for diabetes family history (no, yes), TG (mg/dL, continuous), and HDL-C (mg/dL, continuous). The stratified analyses were presented as a multivariable model to evaluate the association between antioxidant enzyme activities (SOD and CAT) and the T2D risk according to the potential effect modification by age, BMI, physical activity, body fat, visceral fat, HOMA-IR, HOMA-β, TC, HDL-C, LDL-C, and TG.

The cutoff points were <23 kg/m^2^ versus ≥23 kg/m^2^ for BMI, <2 times/week versus ≥3 times/week for physical activity, ≤9% versus >9% for visceral fat, <200 mg/dL versus ≥200 mg/dL for TC, and <150 mg/dL versus ≥150 mg/dL for TG. The other variable biomarkers were below versus above the median values. The cutoff values for the median values were as follows: <45 years versus ≥45 years for age, <31.15% versus ≥ 31.15% for body fat, <1.2725 versus ≥1.2725 for HOMA-IR, ≤70.1645 versus >70.1645 for HOMA-β, ≤54 mg/dL versus >54 mg/dL for HDL-C, and <126 mg/dL versus ≥126 mg/dL for LDL-C.

Furthermore, we investigated the potential synergy between the levels of antioxidant enzyme activities of SOD and CAT, and the T2D risk, according to the below and above median values of the antioxidant enzyme activity. All the statistical data were analyzed using IBM SPSS software version 26.0 (SPSS Inc., Chicago, IL, USA). The level of statistical significance for all tests was set at *p* < 0.05.

## 3. Results

### 3.1. Participants’ Characteristics

Table 1 summarizes the demographic characteristics of 47 patients with T2D and 55 healthy individuals. The T2D group was generally older than the control group (*p* = 0.013), with 41–50 years as the highest proportion. Most participants had no or had a primary level of education, were farmers or salaried employees, and were current smokers. Each group performed physical activities at least five times per week. However, alcohol consumption was similar between the two groups. Approximately 53.2% of the T2D group had a family history of diabetes (*p* = 0.024).

Table 2 shows the anthropometric characteristics of the T2D and control groups. The T2D group exhibited significantly higher levels of weight, SBP, DBP, BMI, AC, WC, HC, WHR, body fat, visceral fat, and trunk fat and significantly lower levels of muscle mass than the control group. Conversely, height did not significantly differ between the two groups.

The characteristics regarding the daily dietary intake presented no statistical difference between the T2D and control groups (Table 2). However, the T2D group tended to have higher intakes of total energy, protein, fat, carbohydrate, cholesterol, and vitamin A and lower intake of zinc than the control group.

Table 3 enumerates the biochemical and clinical variables of the two groups. The T2D group had significantly higher levels of FBG, 2 h BG, HbA1c, HOMA-IR, and TG and significantly lower HOMA-β and HDL-C levels than the control group. Meanwhile, the LDL-C and TC were not significantly different between the two groups. Regarding the antioxidant enzyme activities, the T2D group had statistically lower SOD (*p* = 0.048) and CAT (*p* = 0.030) levels than the control group (Table 3). However, the difference in vitamin A and E status was not statistically significant between the two groups.

### 3.2. Associations of Antioxidant Enzyme Activities and Vitamin Status with T2D Risk

Table 4 shows the associations between antioxidant enzyme activities and T2D risk according to the quartile categories of SOD and CAT. The levels of antioxidant enzymes were inversely associated with T2D risk. The crude ORs of SOD were 4.04 (95% CI: 1.06–15.37; *p* = 0.041) for the lowest quartile when compared with the highest quartile, which was used for reference. After adjusting for the confounders, the result remained statistically significantly associated with T2D risk. For instance, after adjusting for age, sex, diabetes family history, TG, and HDL-C, the ORs of SOD increased significantly to 4.77 (95% CI: 1.01–22.40; *p* = 0.047) in the lowest quartile in comparison with those in the highest quartile. When compared with the participants in the highest quartile of CAT, those in the lowest quartile had crude and multivariable-adjusted ORs of 3.77 (95% CI: 1.17–12.19; *p* = 0.026) and 4.22 (95% CI: 1.07–16.60; *p* = 0.039), respectively.

The relationships between vitamin status and T2D risk across the quartiles of vitamins A and E were investigated using logistic regression analysis (Table 4). Both vitamins A and E showed no clear associations with T2D incidence. The crude and multivariate-adjusted ORs did not exhibit any significant relationships between the vitamin status and the T2D risk. The crude and multivariable-adjusted ORs of vitamin A for T2D were 1.38 (95% CI: 0.44–4.32; *p* = 0.811) and 1.33 (95% CI: 0.33–5.29; *p* = 0.679), respectively, in the lowest quartile when compared with those in the highest quartile. For vitamin E status, the crude and multivariable-adjusted ORs were 0.79 (95% CI: 0.26–2.39; *p* = 0.683) and 1.04 (95% CI: 0.26–4.14; *p* = 0.954), respectively, for those in the lowest quartile.

### 3.3. Associations between Antioxidant Enzyme Activity and T2D Risk Stratified According to Diabetes Risk Factors

The other possible factors in the association between antioxidant enzyme activity and T2D risk were assessed using a stratified analysis (Table 5). Among the participants whose BMI was >23 kg/m^2^, the ORs of SOD in the lowest quartile significantly increased to 7.53 (95% CI: 1.06–53.49) when compared with those in the highest quartile. Furthermore, the SOD quartiles with a percentage of visceral fat of <9% (OR = 68.18, 95% CI: 1.55–1995.89), a HOMA-IR of more than the median (OR = 15.70, 95% CI: 1.81–136.32), and a TC level of >200 mg/dL (OR = 17.15, 95% CI: 1.17–250.01) were significantly associated with T2D risk.

After being stratified by CAT quartiles, the ORs of participants with a physical activity of less than twice a week, a visceral fat of >9%, a TC of >200 mg/dL, and a TG of >150 mg/dL significantly increased to 42.14 (95% CI: 1.17–1516.03), 11.58 (95% CI: 1.17–114.33), 26.73 (95% CI: 2.33–305.59), and 17.68 (95% CI: 1.26–247.73) in the lowest versus the highest quartiles. Moreover, participants with age, body fat percentage, HDL-C level, and LDL-C level that were less than the median, and a HOMA-IR that was more than the median demonstrated significant associations with T2D risk. In addition, younger participants showed a positive association between low CAT activity and T2D risk but not the older ones. Nevertheless, the results in the lowest versus the highest quartiles of these enzyme activities tended to reveal an increased risk for T2D among participants with a low HOMA-β.

### 3.4. Synergetic Effects of Antioxidant Enzyme Activity on T2D Risk

The combined effects of SOD and CAT activities above and below the median were also analyzed. The crude and multivariable-adjusted ORs of T2D with synergistic effects on low antioxidant enzyme activities (SOD and CAT) versus the high levels were elevated by 2.44 fold (95% CI: 0.65–9.13) and 2.34 fold (95% CI: 0.52–10.43), respectively (Table 6). Although the investigation of the effect of the combined activities of SOD and CAT on T2D showed no statistically significant difference, patients with both low levels of SOD and CAT had an increasing trend of T2D risk.

## 4. Discussion

This cross-sectional study found an inverse association between the levels of antioxidant enzyme activities (SOD and CAT) and T2D risk among Thai middle-aged adults living in a rural area. Factors, such as age, BMI, physical activity, body fat, visceral fat, HOMA-IR, TC, HDL-C, LDL-C, and TG might mediate such an association. Nevertheless, vitamin A and vitamin E levels showed no prospective association with an increased T2D risk.

An increase in age, BMI, WC, WHR, body fat percentage, visceral fat percentage, LDL-C level, and TG level and a decrease in lower HDL-C concentrations can predict diabetes incidence [18,19]. Lifestyle patterns consisting of low physical activity, frequent cigarette smoking, excessive alcohol consumption, and a family history of diabetes are also reportedly independent risk factors for T2D [19,20]. In addition, higher levels of blood glucose, cholesterol, and blood pressure are associated with higher levels of insulin resistance and the onset of T2D [21]. The present study presented that these diabetes risk factors are consistent with the previous observations. A possible mechanism of these T2D risk factors could be disturbing of feedback regulation on glucose homoeostasis, inducing endoplasmic reticulum stress, reducing muscle glucose uptake, impaired translocation of glucose transporter 4 and interrupting the phosphorylation of insulin receptors [22].

We found no statistical difference between the T2D and healthy groups in terms of dietary intake. This result may be attributed to the recall bias of each participant or the accuracy of recording by trained staff. However, high total energy intake, excessive carbohydrate consumption, and high dietary cholesterol intake are strongly related to poor glycemic control, insulin resistance, and future T2D risk [23,24,25]. The lower zinc intake correlates with a higher risk for T2D, whereas a higher vitamin A intake is related to poor glucose homeostasis [26,27].

We found significantly higher levels of FBG, 2 h BG, HbA1c, HOMA-IR, and TG in participants with T2D than in the control group. The T2D group also had lower HOMA-β and HDL-C levels. These results are consistent with the previous studies [28]. The proposed mechanisms for T2D development were hypothesized on a defect in the glucose-fatty acid cycle, an excessive accumulation of lipid metabolites (diacylglycerol and ceramide species), a hexosamine biosynthesis pathway and an ectopic lipid accumulation [29].

Low concentrations of SOD and CAT may be linked to T2D through OS-induced hyperglycemia in patients with diabetes. This finding agrees with other studies, which showed decreased levels of SOD and CAT in patients with T2D [30]. Hou et al. (2021) reported a statistically significant decrease in the SOD activity in participants with T2D when compared with that in healthy individuals [5]. Similarly, Lipa et al. (2016) concluded that low serum levels of SOD significantly correlated with the increased risk of T2D and cataract development among individuals with T2D [31]. Other previous studies also reported that patients with T2D have a reduced activity of serum SOD when compared with those without T2D [10,32].

Likewise, Góth et al. (2016) showed significantly lower CAT activities in patients with T2D when compared with those in controls [33]. Low CAT levels can lead to T2D pathogenesis by decreasing insulin secretion and inducing oxidative damage on pancreatic β-cells [3]. A similar trend was observed by Palekar et al. (2016), who observed a significant decrease in CAT levels in participants with T2D when compared with those in controls [34]. Another study by Lipa et al. revealed that the serum CAT levels were significantly lower in participants with T2D than in those without T2D [31].

Decreased levels of SOD and CAT could result from hyperglycemic conditions that lead to glycation; inactivation by crosslinking enzymes; increased lipid peroxidation; elevated susceptibility of these enzymes to free radicals, resulting in the limited potential to detoxify the radicals; and gene mutations of these enzymes [30,34,35]. Moreover, the decreased SOD and CAT levels might be caused by the activation of protein kinase C and nonenzymatic glycosylation, and the loss of cofactors, including Zn^2+^ and Cu^2+^, which are components of these enzymes [5,7]. According to a previous literature review, the downregulation of SOD activity might increase superoxide radicals, leading to CAT inactivation and reduced insulin effectiveness in patients with T2D [5,33].

In contrast, some studies found elevated or unchanged levels of SOD and CAT in patients with T2D. For example, Dworzanski et al. (2020) presented elevated SOD and CAT levels in individuals with T2D, whereas Nwakulite et al. (2021) and Jin et al. (2020) showed no significant increase in SOD and CAT levels in people with T2D when compared with those in people without T2D [11,36,37]. The causation of elevated and unchanged levels of SOD and CAT could be linked to the overexpression of these enzymes to get rid of oxidative attack and peroxidation of polyunsaturated fatty acids in the cell membrane of patients with diabetes so that free radicals are compensated [5]. These discrepancies could be attributed to the longer duration of T2D, diabetes stage, genotype background, different study populations, different sample sizes, and different SOD and CAT analysis methods [3,10,36].

Moreover, our study revealed no clear associations of both vitamins A and E with T2D incidence. These findings are consistent with other studies, which showed no significantly different correlations between the vitamin A and E status and the risk of T2D [16,38,39]. Another explanation might be because of an adequate intake of both vitamins, which was found in our study groups. The medians of both vitamin intakes reached above the Dietary Reference Intake for Thais 2020 [40]. However, controversial reports on changes in vitamin A and E status have been published. Some studies reported decreased or elevated vitamin A and E concentrations in the T2D group when compared with those in the control group [35,39]. A possible explanation for these discrepancies could be the differences in testing methods, study design, region, ethnicity, dietary habits, and disease duration [35,39].

Regarding the BMI, the stratified analysis implied that participants with a higher BMI showed a positive association between the lowest and the highest quartiles of SOD levels, and the risk of T2D; conversely, those with a lower BMI demonstrated a weaker association. This result is in line with the previous findings, which revealed that lower SOD levels are related to the recent onset of T2D among individuals with a higher BMI. These results may occur because of the impaired insulin secretion and pancreatic sufficiency in people which are overweight or obese through an increased secretion of non-esterified fatty acids (NEFAs), which are secreted from adipose tissue. The releasing of high levels of NEFAs will affect the peripheral insulin uptake, leading to the hypothesis of insulin resistance and a decreased SOD level [5,8,35,41,42].

The effect of TC levels on the association between the lowest versus highest quartile of SOD activity and T2D risk is consistent with a previous report. Patients with diabetes and with low SOD concentrations have higher TC levels than healthy individuals. An accumulation of high TC levels in mitochondrial membranes in T2D subjects might result in an increased ROS production and a reduced SOD activity via increased glucokinase, decreased exocytosis of insulin granules, and reduced glucose transporter membrane levels, leading to mitochondrial stress and severe impairment of β-cell [42,43]. However, the relationship between SOD activity and T2D risk among individuals with a percentage of visceral fat of <9% remains uninvestigated.

Further, a low CAT activity was associated with T2D risk in participants with a higher percentage of body composition (i.e., BMI, waist, WHR, and visceral fat) or lower physical activity. A few studies have reported the effect of these factors on the association between CAT activity and T2D risk [34,44]. However, there are some plausible explanations consisting of increased adipose tissue leading to an elevated systemic release of resistin, raised circulating of interleukins and cytokines, and impaired insulin receptors. Consequently, β-cell compensation could not work efficiently for the decreased insulin sensitivity, which resulted in inducing OS and decreasing CAT activity-linked diabetes [45].

In the present study, CAT–T2D risk relationship was strong among individuals with high HOMA-IR and TC levels, consistent with the previous studies [5,33]. It can be explained as follows: a decreased CAT activity is related to both parameters due to the accumulation of the synergistic toxic effect between glucotoxicity, inducing insulin resistance and lipotoxicity from elevating levels of cholesterol. They affect and alter insulin secretion and insulin gene transcription. Therefore, these processes have been suggested as one of the mechanisms in T2D development [46]. Similarly, other studies have shown that through endoplasmic reticulum stress and lipotoxicity, high TG concentrations and low HDL-C levels might increase T2D risk in participants with low CAT levels [5,33,47].

Our study has also shown a low percentage of body fat or low LDL-C levels in participants with low CAT activity. These associations are inconsistent with the previous studies [33,34]. However, the relationship between CAT and T2D risk on the age subgroup analyses contradicted the previous findings, which revealed that older people have a higher T2D risk than the younger ones [31,35]. The proposed reason for the conflicting results on CAT activity could be a cellular response as a compensatory adaptation to OS during the downregulation of CAT, genetic mechanisms and body fat accretion among individuals, and a disturbance in the protein tyrosine kinase and protein tyrosine phosphatase in cell signaling pathways during aging on impaired CAT activity [48].

Nevertheless, the mechanism of CAT–T2D association with these several factors in this stratified analysis is not fully understood. The analysis did not reveal a clear difference in HOMA-β in the association between SOD and CAT quartiles and T2D risk.

The combined low concentrations of the antioxidant enzyme activities (SOD and CAT) may be related to T2D pathogenesis. Our research conforms to the knowledge on the susceptibility of red cell membranes to free-radical overproduction and dysfunctional enzyme activities, including SOD and CAT. These phenomena lead to β-cell dysfunction and insulin resistance, which are the major mechanisms of T2D occurrence [34].

This study has some limitations. First, the data used for investigation were only obtained from the Thai population in Nakhon Rachasima, and the sample size was small, resulting in widened CIs. A larger sample size from various regions is needed to verify the association between the antioxidant defense strategies and T2D risk. Second, regarding the collection of data on semi-FFQ, cigarette smoking, and alcohol intake, recall bias is possible. Third, the stability of serum in our study was not directly measured.

## 5. Conclusions

Unlike vitamin A and vitamin E status, the levels of antioxidant enzyme activities (SOD and CAT) were inversely associated with an increased T2D risk among rural Thais. Age, BMI, physical activity, body fat, visceral fat, HOMA-IR, TC, HDL-C, LDL-C, and TG might mediate the effect of the circulatory levels of SOD and CAT on T2D. The reduced SOD and CAT levels may be used as a screening tool for monitoring T2D risk. Additionally, the measurement of these biomarkers might be beneficial in T2D prevention and management. Further research with a large sample size is needed to confirm these associations and elucidate the possible mechanisms of antioxidant enzyme activities in modulating T2D pathogenesis.

## Figures and Tables

**Table 1:** Demographic characteristics of control group and type 2 diabetic (T2D) patients.

Variables	Control Group (*n* = 55)	T2D Group (*n* = 47)	*p* Value
Gender (n, %)			0.579
Male	18 (32.7)	13 (27.7)	
Female	37 (67.3)	34 (72.3)	
Age (years)	44 (35, 60)	48 (35, 66)	0.013
Age classification (n, %)			0.019
≤40	13 (23.6)	6 (12.8)	
41–50	34 (61.8)	23 (48.9)	
≥51	8 (14.5)	18 (38.3)	
Education level (n, %)			0.242
Primary school or below	43 (78.2)	39 (83.0)	
Secondary school	11 (20.0)	5 (10.6)	
Vocational college or above	1 (1.8)	3 (6.4)	
Occupation (n, %)			0.648
Agriculture	21 (38.2)	21 (44.7)	
Salaried	25 (45.5)	21 (44.7)	
Others	9 (16.4)	5 (10.6)	
Cigarette smoking (n, %)			0.367
Never and former smokers	16 (29.1)	10 (21.3)	
Current smokers	39 (70.9)	37 (78.7)	
Alcohol intake (n, %)			0.761
No	23 (41.8)	22 (46.8)	
Yes	32 (58.2)	25 (53.2)	
Physical activity (n, %)			0.292
Rarely/never	19 (34.5)	9 (19.1)	
1–2 times/week	6 (10.9)	6 (12.8)	
3–4 times/week	5 (9.1)	5 (10.6)	
≥5 times/week	25 (45.5)	27 (57.4)	
Family history of diabetes (n, %)			0.024
No	39 (70.9)	22 (46.8)	
Yes	16 (29.1)	25 (53.2)	

Data are expressed as median (min, max) or number (percentage) as appropriate. Abbreviation: T2D; type 2 diabetes. Comparisons of category variables between groups were analyzed by chi-square test and comparisons of continuous variables between groups were analyzed by Mann–Whitney’s test.

**Table 2:** Anthropometric measures and daily dietary intakes of control group and T2D patients.

Variables	Control Group (*n* = 55)	T2D Group (*n* = 47)	*p* Value
Weight (kg)	57.95 (39.25, 86.65)	68.25 (41.60, 108.65)	0.001
Height (cm)	157 (143, 174)	156 (146, 172)	0.144
SBP (mmHg)	120 (91, 158)	129 (99, 181)	0.039
DBP (mmHg)	76 (49, 96)	79 (51, 98)	0.025
BMI (kg/m^2^)	24.02 (17.34, 30.96)	27.23 (17.52, 43.80)	<0.001
AC (cm)	80.50 (69.00, 101.50)	93.00 (73.10, 121.30)	<0.001
WC (cm)	80.00 (65.00, 102.50)	89.00 (70.00, 115.00)	<0.001
HC (cm)	93.00 (77.50, 110.00)	98.00 (77.00, 127.50)	0.019
WHR	0.85 (0.72, 1.00)	0.90 (0.74, 1.01)	<0.001
Body fat (%)	28.30 (16.40, 40.00)	34.60 (6.80, 44.80)	<0.001
Visceral fat (%)	7.50 (2.00, 33.10)	11.50 (2.00, 40.50)	<0.001
Trunk fat (%)	20.60 (9.20, 33.10)	27.50 (5.30, 42.20)	0.001
Muscle mass (%)	25.70 (21.80, 33.70)	23.50 (14.60, 36.70)	<0.001
Total energy (kcal/day)	2022.6 (792.4, 7589.5)	2304.6 (566.5, 5910.9)	0.658
Protein (g/day)	63.1 (27.3, 291.8)	65.8 (15.3, 203.9)	0.913
Fat (g/day)	51.9 (14.2, 254.0)	54.5 (7.8, 195.9)	0.849
Carbohydrate (g/day)	316.6 (109.1, 1187.0)	373.6 (76.1, 952.2)	0.562
Dietary fiber (g/day)	9.5 (2.6, 41.8)	10.3 (1.6, 47.8)	0.970
Cholesterol (mg/day)	256.4 (11.1, 1246.0)	263.9 (1.3, 626.0)	0.562
Vitamin A (µg/day)	519.5 (78.3, 2798.4)	666.0 (100.1, 3429.5)	0.460
Vitamin E (mg/day)	0.2 (0.0, 1.2)	0.2 (0.0, 1.3)	0.956
Iron (mg/day)	11.8 (5.5, 44.3)	13.4 (3.2, 48.4)	0.480
Zinc (mg/day)	2.2 (0.3, 12.2)	2.1 (0.2, 7.4)	0.655

Abbreviations: T2D; type 2 diabetes; SBP, systolic blood pressure; DBP, diastolic blood pressure; BMI, body mass index; AC, abdominal circumference; WC, waist circumference; HC, hip circumference; WHR, waist-to-hip ratio.

**Table 3:** Clinical profiles, antioxidant enzyme activities, and vitamin status of control group and T2D patients.

Variables	Control Group (*n* = 55)	T2D Group (*n* = 47)	*p* Value
FBG (mg/dL)	87 (65, 98)	110 (69, 261)	<0.001
2-h BG (mg/dL)	103 (42, 176)	207 (85, 480)	<0.001
HbA1c (%)	5.3 (3.3, 6.1)	5.9 (4.5, 10.7)	<0.001
HOMA-IR	1.16 (0.01, 2.72)	1.60 (0.15, 17.81)	<0.001
HOMA-β	86.17 (0.66, 341.47)	44.55 (7.81, 188.62)	<0.001
TG (mg/dL)	102 (43, 601)	148 (64, 694)	0.002
HDL-C (mg/dL)	56 (19, 118)	52 (3, 114)	0.027
LDL-C (mg/dL)	122 (60, 233)	126 (54, 303)	0.987
Total cholesterol (mg/dL)	203 (9, 335)	206 (77, 368)	0.917
SOD (%)	82.35 (16.67, 164.71)	77.93 (11.76, 123.53)	0.048
CAT (nmol/min/mL)	16.63 (0.23, 82.70)	6.56 (0.23, 58.79)	0.030
Vitamin A (mg/L)	0.62 (0.28, 0.94)	0.64 (0.28, 1.10)	0.867
Vitamin E (mg/L)	10.43 (6.47, 15.60)	10.83 (4.32, 17.92)	0.951

Abbreviations: T2D; type 2 diabetes; FBG, fasting blood glucose; 2-h BG, 2-h blood glucose after 75 g oral glucose tolerance test; HbA1c, *glycated hemoglobin*; HOMA-IR, homeostatic model assessment of insulin resistance; HOMA-β, homeostatic model assessment of β-cell function; TG, triglyceride; HDL-C, high-density lipoprotein cholesterol; LDL-C, low-density lipoprotein cholesterol; SOD, superoxide dismutase; CAT, catalase.

**Table 4:** Odds ratios (ORs) and 95% confidence intervals (CIs) according to quartiles of antioxidant enzyme activities and vitamin status for T2D.

Variables	No. of T2D (%)	ORs (95% CIs)					
		Crude	*p* Value	Model 1	*p* Value	Model 2	*p* Value
Antioxidant enzyme activities							
Quartiles of SOD (%)							
Q1	14 (51.9)	4.04 (1.06–15.37)	0.041	4.46 (1.09–18.23)	0.037	4.77 (1.01–22.40)	0.047
Q2	12 (46.2)	3.21 (0.84–12.35)	0.089	3.08 (0.76–12.42)	0.113	3.05 (0.65–14.29)	0.157
Q3	16 (55.2)	4.61 (1.22–17.34)	0.024	4.53 (1.15–17.87)	0.031	6.12 (1.30–28.83)	0.022
Q4	4 (21.1)	1.00 (reference)		1.00 (reference)		1.00 (reference)	
Quartiles of CAT (nmol/min/mL)							
Q1	17 (68.0)	3.77 (1.17–12.19)	0.026	4.38 (1.24–15.48)	0.022	4.22 (1.07–16.60)	0.039
Q2	11 (42.3)	1.30 (0.42–4.02)	0.645	1.46 (0.44–4.77)	0.527	1.27 (0.34–4.72)	0.712
Q3	10 (38.5)	1.11 (0.35–3.46)	0.856	1.00 (0.30–3.32)	0.996	0.93 (0.24–3.55)	0.917
Q4	9 (36.0)	1.00 (reference)		1.00 (reference)		1.00 (reference)	
Vitamin status							
Quartiles of vitamin A (mg/L)							
Q1	14 (56.0)	1.38 (0.44–4.32)	0.811	1.43 (0.42–4.82)	0.560	1.33 (0.33–5.29)	0.679
Q2	10 (38.5)	0.68 (0.21–2.12)	0.509	0.66 (0.19–2.20)	0.502	0.62 (0.16–2.40)	0.490
Q3	12 (44.4)	0.87 (0.28–2.66)	0.572	0.91 (0.285–2.95)	0.886	0.81 (0.23–2.87)	0.750
Q4	11 (47.8)	1.00 (reference)		1.00 (reference)		1.00 (reference)	
Quartiles of Vitamin E (mg/L)							
Q1	12 (48.0)	0.79 (0.26–2.39)	0.683	0.81 (0.24–2.72)	0.744	1.04 (0.26–4.14)	0.954
Q2	11 (42.3)	1.17 (0.38–3.56)	0.777	1.54 (0.46–5.14)	0.477	2.11 (0.49–9.01)	0.313
Q3	13 (52.0)	0.85 (0.28–2.59)	0.777	0.81 (0.24–2.70)	0.732	0.85 (0.20–3.52)	0.827
Q4	11 (44.0)	1.00 (reference)		1.00 (reference)		1.00 (reference)	

SOD: Q1, ≤64.7100; Q2, 64.7101–83.3300; Q3, 83.3301–105.8800; Q4, ≥105.8801. CAT: Q1, ≤2.5875; Q2, 2.5876–11.7100; Q3, 11.7101–32.2500; Q4, ≥32.3501. Vitamin A: Q1, ≤0.4950; Q2, 0.4951–0.6200; Q3, 0.6201–0.8100; Q4, ≥0.8101. Vitamin E: Q1, ≤8.7250; Q2, 8.7251–10.4300; Q3, 10.4301–11.9250; Q4, ≥11.9251. Crude: no adjustment. Model 1: adjusted odds ratios were estimated for age and gender. Model 2: based on model 1 and additionally adjusted for family history of diabetes, TG and HDL-C. Abbreviations: ORs, odds ratios; CIs, confidence intervals; Q, quartile; T2D; type 2 diabetes; SOD, superoxide dismutase; CAT, catalase; TG, triglyceride; HDL-C, high-density lipoprotein cholesterol.

**Table 5:** Odds ratios and 95% confidence intervals of T2D between highest (Q4) and lowest quartiles (Q1) of superoxide dismutase and catalase in relation to strata for various factors.

Variables	No. of T2D (%)	SOD (%)		CAT (nmol/min/mL)	
		Adjusted		Adjusted	
		ORs	CIs	ORs	CIs
Age (years) ^a^					
Low	17 (36.2)	2.31	(0.13–40.50)	24.91	(1.46–422.83) *
High	30 (54.5)	6.61	(0.89–48.97)	1.67	(0.30–9.13)
BMI (kg/m^2^)					
Low	12 (26.1)	1.06	(0.03–31.78)	3.26	(0.27–38.32)
High	35 (62.5)	7.53	(1.06–53.49) *	5.14	(0.66–39.55)
Physical activity					
Low	15 (37.5)	5.36	(0.25–114.86)	42.14	(1.17–1516.03) *
High	32 (51.6)	4.96	(0.68–36.28)	1.39	(0.26–7.50)
Body fat (%)					
Low	16 (31.4)	5.67	(0.35–91.72)	10.83	(1.07–109.11) *
High	31 (60.8)	3.84	(0.45–32.33)	1.46	(0.17–12.39)
Visceral fat (%)					
Low	14 (28.0)	68.18	(1.55–1995.89) *	1.75	(0.21–14.13)
High	33 (63.5)	4.09	(0.56–29.68)	11.58	(1.17–114.33) *
HOMA-IR					
Low	13 (25.5)	0.56	(0.03–9.01)	2.59	(0.32–21.13)
High	34 (66.7)	15.70	(1.81–136.32) *	10.43	(1.03–105.60) *
HOMA-β					
Low	33 (64.7)	1.83	(0.23–14.59)	2.28	(0.32–15.15)
High	14 (27.5)	1.30	(0.19–8.53)	1.25	(0.04–37.31)
TC (mg/dL)					
Low	23 (45.1)	2.08	(0.20–20.96)	0.68	(0.08–5.62)
High	24 (47.1)	17.15	(1.17–250.01) *	26.73	(2.33–305.59) *
HDL-C ^b^ (mg/dL)					
Low	27 (54.0)	2.68	(0.26–27.74)	12.40	(1.31–117.27) *
High	20 (38.5)	6.58	(0.50–85.64)	2.08	(0.38–11.39)
LDL-C (mg/dL)					
Low	21 (42.0)	3.71	(0.31–43.45)	16.18	(1.04–247.72) *
High	26 (50.0)	7.43	(0.52–105.61)	6.51	(0.82–51.44)
TG ^c^ (mg/dL)					
Low	24 (38.7)	4.58	(0.34–61.18)	2.74	(0.37–20.24)
High	23 (57.5)	8.75	(0.75–101.09)	17.68	(1.26–247.73) *

The various factors including status of age: low, <45 years; high, ≥45 years; BMI: low, <23 kg/m^2^; high, ≥23 kg/m^2^; physical activity: low, <2 times/week; high, ≥3 times/week; body fat: low, <31.15 %; high, ≥31.15 %; visceral fat: low, ≤9; high, >9; HOMA-IR, low <1.2725; high, ≥1.2725; HOMA-β: low, ≤70.1645; high, >70.1645; total cholesterol: low, <200 mg/dL; high, ≥200 mg/dL; HDL-C: low, ≤54 mg/dL; high, >54 mg/dL; LDL-C: low, <126 mg/dL; high, ≥126 mg/dL; TG: low, <150 mg/dL; high, ≥150 mg/dL. Adjusted model: adjusted odds ratios were estimated for age, gender, family history of diabetes, TG and HDL-C. ^a^ The model was not adjusted for age. ^b^ The model was not adjusted for HDL-C. ^c^ The model was not adjusted for TG. Asterisks denote significant differences between highest and lowest quartiles (* *p* < 0.05). Abbreviations: ORs, odds ratios; CIs, confidence intervals; T2D; type 2 diabetes; SOD, superoxide dismutase; CAT, catalase; BMI, body mass index; HOMA-IR, homeostatic model assessment of insulin resistance; HOMA-β, homeostatic model assessment of β-cell function; TC, total cholesterol; HDL-C, high-density lipoprotein cholesterol; LDL-C, low-density lipoprotein cholesterol; TG, triglyceride.

**Table 6:** Combinations of antioxidant enzyme activities of SOD and CAT with risk of T2D incidence.

Variables	Subjects (*n*)	T2D, *n* (%)	Crude	Adjusted
			ORs (95% CIs)	ORs (95% CIs)
Low SOD Low CAT	18	12 (66.7)	2.44 (0.65–9.13)	2.34 (0.52–10.43)
Low SOD High CAT	30	9 (30.0)	0.52 (0.161–1.70)	0.50 (0.13–1.87)
High SOD Low CAT	34	16 (47.1)	1.15 (0.37–3.50)	1.39 (0.39–4.99)
High SOD High CAT	20	9 (45.0)	1.00 (reference)	1.00 (reference)

Low and high of antioxidant enzyme activities of SOD (83.3300 %) and CAT (11.7100 nmol/min/mL) were defined as median values. Crude: no adjustment. Adjusted model: adjusted odds ratios were estimated for age, gender, family history of diabetes, TG and HDL-C. Abbreviations: ORs, odds ratios; CIs, confidence intervals; T2D; type 2 diabetes; SOD, superoxide dismutase; CAT, catalase.

## Data Availability

The data of this study are available from the first and corresponding author on request.
